# Redox State of Human Serum Albumin in Multiple Sclerosis: A Pilot Study

**DOI:** 10.3390/ijms232415806

**Published:** 2022-12-13

**Authors:** Margret Paar, Katharina Seifried, Gerhard Cvirn, Arabella Buchmann, Michael Khalil, Karl Oettl

**Affiliations:** 1 Division of Medicinal Chemistry, Otto Loewi Research Center, Medical University of Graz, Neue Stiftingtalstraße 6, 8010 Graz, Austria; 2 Department of Neurology, Medical University of Graz, 8036 Graz, Austria

**Keywords:** human serum albumin, human mercaptalbumin, human nonmercaptalbumin, human cerebrospinal fluid albumin, albumin redox state, multiple sclerosis, biomarker, cerebrospinal fluid (CSF), serum

## Abstract

Like in many other pathologies, oxidative stress is involved in the development of neurodegenerative disorders. Human serum albumin (HSA) is the main protein in different body fluids including cerebrospinal fluid (CSF). By its redox state in terms of cysteine-34, albumin serves as marker for oxidative burden. We aimed to evaluate the redox state of HSA in patients with multiple sclerosis in serum and CSF in comparison to controls to identify possible correlations with disease activity and severity. Samples were stored at −70 °C until analysis by HPLC for the determination of albumin redox state in terms of the fractions of human mercaptalbumin (HMA), human nonmercaptalbumin1 (HNA1), and human nonmercaptalbumin2 (HNA2). Albumin in CSF showed significantly higher fractions of the reduced form HMA and decreased HNA1 and HNA2. There was no difference between albumin redox states in serum of patients and controls. In CSF of patients HNA2 showed a trend to higher fractions compared to controls. Albumin redox state in serum was associated with physical disability in remission while albumin redox state in CSF was related to disease activity. Thus, albumin redox state in serum and CSF of patients in relation to disease condition merits further investigation.

## 1. Introduction

Oxidative challenges have been reported to occur during the development of neurodegenerative diseases in general, including in multiple sclerosis (MS) [1,2,3]. MS is a chronic neuro-inflammatory disease of the brain and spinal cord that represents a common cause of physical and cognitive disability in young adults, especially women [4]. The cause of MS is still unknown, but it is believed that genetic predisposition and environmental factors such as infections, nutrition, smoking, and vitamin D levels are driving MS pathogenesis [2,5]. Although CD4+ T cells may play a central role in MS pathogenesis, increasing evidence suggests a broad involvement of activated cells from both the innate and adaptive immune systems [5]. In addition, oxidative stress responses may facilitate brain-tissue damage and promote pathophysiological processes leading to neurodegeneration [6,7]. 

The role of oxidative stress as cause or consequence of pathological changes is not yet fully understood. Different markers can be used to monitor the oxidative burden in different body compartments. In this respect, human serum albumin (HSA) is a quite interesting molecule for MS as well as for oxidative stress in general. HSA is the main protein in multiple body fluids, e.g., vitreous humor and cerebrospinal fluid (CSF) [8,9]. However, its concentration varies considerably. In CSF, the concentration is between 150 and 350 mg/L which is below one percent of serum concentration (35–55 g/L). Despite some studies that report the synthesis of albumin in microglia in cell culture [10], albumin in CSF is considered to mainly originate from the liver via serum [11,12]. The relationship of albumin concentration in CSF vs. serum, the albumin quotient (Q_Alb_), serves as a measure for the leakage of the blood–brain barrier (BBB) [8]. The redox state of albumin in terms of cysteine-34 (Cys-34), on the other hand, is an indicator for redox balance as albumin occurs in three different redox states concerning Cys-34: (i) human mercaptalbumin (HMA), the reduced form with a free thiol group on Cys-34; (ii) human non-mercaptalbumin-1 (HNA1) oxidized, with a disulfide bond, to cysteine, homocysteine, or glutathione; and (iii) human non-mercaptalbumin-2 (HNA2) with cys-34 oxidized to sulfinic or sulfonic acid [13]. Whereas HNA2 is described as an irreversibly oxidized form, HMA and HNA1 are reversibly convertible depending on the concentration of “small” thiols and disulfides such as cysteine/cystine or reduced and oxidized glutathione (GSH/GSSG) in the environment of the albumin molecule. The redox state of HSA in health and different pathologies has been described [14,15,16,17]. However, data concerning the redox state of albumin in CSF are scarce.

It has been reported that the redox state of albumin in CSF differs from that in serum and that in Alzheimer’s disease (AD), CSF albumin is significantly shifted to the oxidized state [14]. Data concerning the redox state of albumin in MS are still missing. Therefore, the aim of this study was to determine the albumin redox state in MS patients and controls in CSF and serum to elucidate possible differences and/or correlations with disease activity and severity.

## 2. Results

### 2.1. Albumin Redox State in Relation to Demographic and Laboratory Data

We evaluated albumin redox state in serum and CSF of 20 MS patients and 21 controls (Table 1). The patient group consisted of 17 relapsing–remitting MS (RRMS) and 3 primary-progressive MS (PPMS) patients. Further characteristics of the groups and routine diagnostic parameters are given in Table 1 and Table 2, respectively.

**Figure 1:**
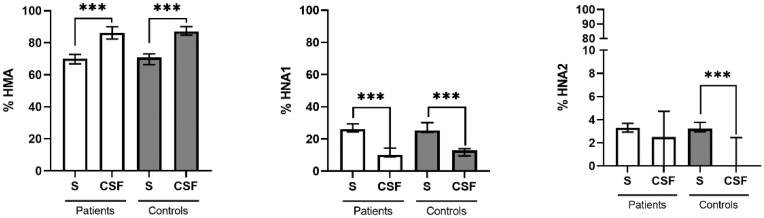
Redox state of albumin in serum versus CSF of MS patients (RRMS and PPMS) and controls. Fractions of HMA (left panel), HNA1 (middle panel), and HNA2 (right panel) in serum (S) and cerebrospinal fluid (CSF) of MS patients (white bars) and controls (gray bars). Data are presented as medians and interquartile ranges. Differences between S and CSF were assessed by Wilcoxon matched-pairs signed rank test. ***, *p* < 0.001.

The redox state of serum and CSF albumin was evaluated in all patients and controls. No significant differences between patients and controls were found for HMA and HNA1 in either compartment (Table 2). In CSF, there was a trend of higher HNA2 fraction in patients compared to controls. However, significant differences of albumin redox states between serum and CSF were observed in both groups. HMA fractions were significantly higher and HNA1 fractions significantly lower in CSF compared to serum. HNA2 was significantly lower in the control but not the patient group (Figure 1). In the control group HNA2 in CSF was positively correlated with HNA2 in serum (see below). No other correlations were observed between albumin redox states in serum and CSF in the entire cohort, or when separately analyzing patients and controls (HMA: r = 0.072, *p* = 0.76; r = 0.32, *p* = 0.164; r = 0.20, *p* = 0.20; HNA1: r = 0.264, *p* = 0.26; r = 0.331, *p* = 0.14; r = 0.225, *p* = 0.16; HNA2: r = −0.121, *p* = 0.61; **r = 0.495**, ***p* = 0.02**; r = 0.195, *p* = 0.22).

In the combined analysis of patients and controls, we found a significant correlation of serum HNA2 with the age at sampling (r = 0.417, *p* = 0.007), which was mainly driven by the control group (r = 0.605, *p* = 0.003). No such correlation was present when analyzing solely the MS patient group (r = 0.241, *p* = 0.31). In CSF of patients and controls, together, we found a moderate linear correlation of HMA with the age at sampling (r= −0.324, *p* = 0.04). In the control group there was also a correlation of HNA2 in CSF with age (r = 0.524, *p* = 0.01). Likewise, these correlations were not observed when examining only the patient group (HMA: r = −0.289, *p* = 0.22; HNA2: r = 0.029, *p* = 0.90). No significant correlation of HNA1 with the age at sampling was observed in either of the groups. Detailed data on correlations of albumin redox state with the age at sampling are given in Table 3.

Albumin redox state in serum of patients and the combined group of patients and controls was associated with lactate in CSF. Negative correlations were observed for serum HMA, positive correlations for serum HNA1 and HNA2 with lactate in CSF. No such correlations were observed when analyzing solely the control group. Detailed data on age-corrected correlations of albumin redox state in serum with lactate in CSF are given in Table 4.

### 2.2. Albumin Redox State in Relation to Disease Severity in MS

**Figure 2:**
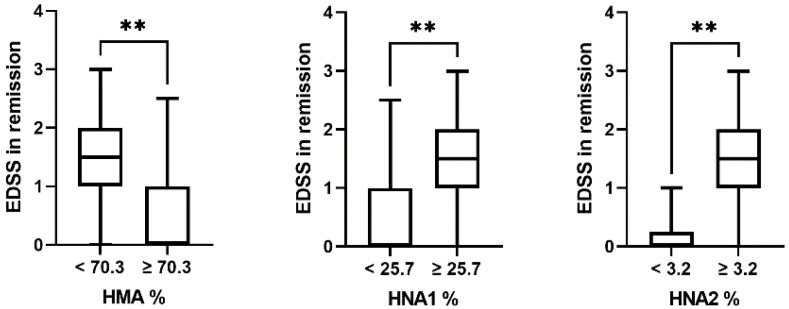
Association of albumin redox state in serum with physical disability in remission. Patients (RRMS and PPMS) were divided into two groups with albumin redox fractions < or ≥ the median serum fraction of HMA (left panel), HNA1 (middle panel), or HNA2 (right panel) in patients and controls. n of the patients in the respective groups: HMA, 11/9; HNA1, 7/13; HNA2, 10/10. **, *p* < 0.01 by Mann–Whitney U tests.

Within our patient group, albumin redox state in serum but not in CSF was associated with the extent of physical disability in remission. Patients with lower HMA and higher HNA1 and HNA2 serum levels had significantly higher Expanded Disability Status Scale (EDSS) scores in remission compared to patients with higher HMA and lower HNA1 serum levels (Figure 2). Furthermore, EDSS in remission correlated significantly with HMA and HNA1 in serum (r = −0.447, *p* < 0.05; r = 0.452, *p* < 0.05) but not with HNA2 in serum (r = 0.417, *p* = 0.07). However, these correlations were lost when correcting for age (HMA: r = −0.339, *p* = 0.16; HNA1: r = 0.331, *p* = 0.17).

**Figure 3:**
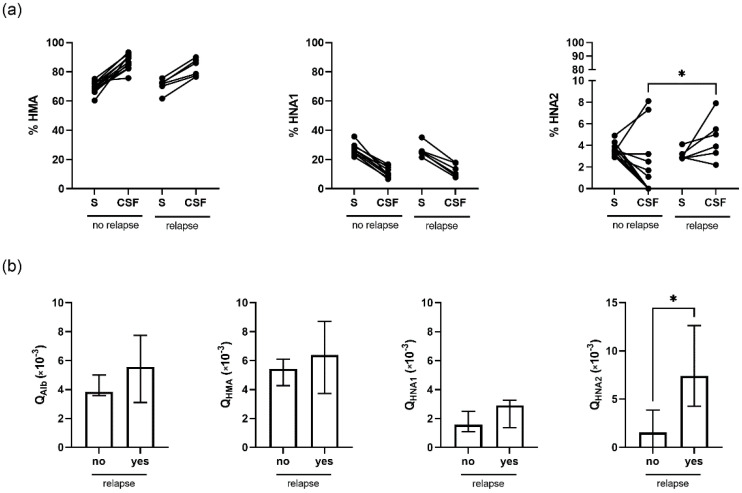
Association of albumin redox state and disease activity in RRMS. (**a**) HMA, HNA1, and HNA2 fractions were determined in serum (S) and CSF of 6 patients with (relapse) and 11 patients without relapse (no relapse) within the last 14 days before sampling. (**b**) Albumin-quotient (Q_Alb_) and HMA-, HNA1-, and HNA2-quotients (Q_HMA_, Q_HNA1_, Q_HNA2_) of patients with or without relapse within the last 14 days before sampling. *, *p* < 0.05 by Mann–Whitney U test.

Albumin redox state in CSF was related to disease activity in RRMS. Six patients suffered a relapse within the last 2 weeks before sampling. In these patients, HNA2 fraction was significantly elevated in CSF compared to patients with a longer time interval between last relapse and sampling (*p* = 0.04) (Figure 3a, right panel). In addition, the relationship of absolute HNA2 levels in CSF to serum, Q_HNA2_, was elevated in the RRMS patient group with relapse within the last two weeks prior to sampling (*p* = 0.01) (Figure 3b, right panel).

## 3. Discussion

Oxidative burden has been suggested to play an important role in neurodegenerative disorders. Redox states of HSA could be representative of the environment in which albumin exists. We could confirm the results of earlier reports that HSA in CSF appears mainly in the reduced form HMA [14,18]. In the present study we demonstrate that the fraction of HMA was higher in CSF compared to serum and HNA1 was lower in every single case in controls and MS patients. In contrast, the fraction of HNA2 in CSF was lower in most cases but also higher HNA2 fractions were observed in subjects of both groups.

Several studies have reported lower blood albumin levels in MS patients compared to controls (recently reviewed in a meta-analysis study [19]). However, as for other variables, results of different groups are quite divergent. We could not find lower serum albumin in our patient group.

There are (only) two other studies reporting albumin redox state in terms of HMA, HNA1, and HNA2 in CSF. One investigated AD patients and controls [14], the other investigated two patient groups of different age with orthopedic disorders [18]. Our results for serum in the control group are in good agreement with both studies: Costa et al. [14] report 65.5/32.2/2.6 and Matsuyama et al. [18] 76.4/22.0/1.6 %HMA/%HNA1/%HNA2, respectively. The lower fraction of HMA and the higher fraction of HNA1 in [14] may be attributed to the higher age of the control subjects. For control CSF 86.4/10.0/3.8 and 93.0/6.7/0.3 %HMA/%HNA1/%HNA2 are reported in [14] and [18], respectively. HMA and HNA1 of [14] are in good agreement with our results while for HNA2 we have a good accordance with [18]. In total, all three studies give comparable results concerning albumin redox state in serum and CSF. 

The conversion of HMA to HNA1 is reversible and the ratio depends on the environment of the albumin molecule in terms of the concentrations of thiols such as cysteine or GSH, and disulfides such as cystine or GSSG. Although it has been reported that microglia may synthesize albumin in cell culture, the significance thereof is not yet established [10]. HSA in CSF is thought to result as filtrate from serum. Given the high dynamicity and turnover of CSF, the high fraction of HMA is surprising. However, it should be mentioned that the rate constant of the reduction of HNA1 to HMA by reaction with a small thiol is quite high compared to the rate constant for the formation of disulfide by reaction with cystine or other disulfides [20].

Several studies report the total thiol content and only a few the disulfide content of CSF in MS patients and controls. However, the methods applied and the results are quite divergent and data concerning the ratio of thiols and disulfides are scarce. Nanomolar [21] and micromolar concentrations [22,23] of GSH in CSF have been reported. In some cases, the reduced form predominates in accordance with the high fraction of HMA. Another study reports higher disulfides compared to thiols [23]. 

An increase in HNA2 indicates the occurrence of reactive species, despite the high levels of HMA. On the other hand, the loss of HNA2 in CSF compared to serum is perplexing as the oxidation of Cys-34 to the sulfinic and sulfonic acid form is thought to be irreversible. The data concerning the slight reduction of HNA2 by astrocytes in cell culture does not appear to fully explain the loss of HNA2 [18]. Perhaps other cell types surrounded by CSF are capable of reducing HNA2 in vivo. Degradation of HNA2 by cells expressing GP18/30, receptors for modified albumin [24], is conceivable as well. Therefore, the expression levels of albumin receptors in cells in contact with CSF should be assessed. Another possible explanation for low HNA2 levels in CSF could be that HNA2 is already hindered on passing the BBB by a hitherto unknown mechanism. Further studies are needed to unravel this interesting aspect.

Based on previous reports, one can expect a correlation of serum HMA and HNA1 with age in healthy subjects, while no such correlation might be present with HNA2 [18,25]. For CSF Matsuyama et al. reported no age-related changes in albumin redox state [18]. In contrast, we observed a significant correlation of age with serum HNA2 and CSF HMA and HNA2, when patients and controls were evaluated together. However, in the patient group alone there was no significant correlation of age with albumin redox state in serum and CSF. This is an interesting finding and could possibly be explained by an altered albumin redox state in CSF and serum in patients with a chronic neurological disease such as MS, which in turn could mask the association of the albumin redox state with age seen in control subjects. Another interesting finding was the correlation of albumin redox state in serum with lactate in CSF. This merits further investigation, as our data do not provide an explanation for it.

There were no differences in the HSA redox state between patients and controls in serum. Furthermore, the fractions of HMA and HNA1 in CSF were essentially identical in both groups. Only HNA2 showed a trend to increased values in the patient group. Although not significant, this is in agreement with a decreased antioxidative capacity in CSF of MS patients [26]. While HNA2 in CSF of the control group was significantly lower than in serum, in our MS patients HNA2 fractions in CSF and serum were in a similar range. In contrast, remarkably high fractions of HNA2 in CSF have been found in a group of AD patients indicating the susceptibility of HSA to oxidation in CSF [14].

Connections of HSA redox state with the disease pattern in MS were found in serum and in CSF. Remarkably, higher physical disability, indicated by higher EDSS in remission, was associated with lower HMA and higher HNA1 and HNA2 in serum and not in CSF. However, in RRMS patients who suffered a relapse within the last 14 days before sample collection, HNA2 in CSF was significantly higher compared to patients with stable disease within this time interval. 

The current study had some limitations. Our data are based on a small patient cohort and it is necessary to confirm our findings in a larger group. Furthermore, most of the patients were in early stages of disease and had a relapsing–remitting disease course, whereas only three patients had primary progressive MS. This limits the comparison of albumin redox states in serum and CSF between patients with different disease phenotypes. Therefore, it would be especially interesting to investigate albumin redox state in serum and CSF from a higher number of patients with progressive disease in a longitudinal prospective study. Although the availability of CSF material is limited, the knowledge concerning HSA characteristics is also of interest with the background of albumin infusion as part of therapies for neurodegenerative disorders such as AD [27]. To obtain a full picture of redox processes in CSF the determination of albumin redox state should be combined with the quantification of small thiols and disulfides. 

In summary, our data confirm the view that oxidative processes are involved in the progress of MS. It is quite interesting that HNA2 can be increased while the thiol/disulfide ratio is shifted to the reduced side. This happens in an environment that is very dynamic and presents HSA as a fascinating analyte for the investigation of redox processes in CSF. Our results provide a reliable basis for future studies with larger patient cohorts to further investigate the clinical significance of altered albumin redox fractions in patients with MS or the suitability as prognostic markers.

## 4. Materials and Methods

This study was approved by the ethics committee of the Medical University of Graz, Austria (ethical approval number: 31-432 ex 18/19).

### 4.1. Study Design and Study Patients

Twenty patients aged 18–52 years (median: 33 years, Q1–Q3: 27–46 years) at time of sampling with a confirmed diagnosis of multiple sclerosis according to McDonald’s 2017 criteria [28] were enrolled in the study. The control group consisted of 21 “symptomatic controls” according to Teunissen et al. [29], aged 18–59 years (median: 36 years, Q1–Q3: 29–42 years). These are patients presenting with neurological symptoms (such as paresthesia or signs of paralysis), where further diagnostic evaluation cannot find any pathological correlates. 

Exclusion criteria for MS patients and controls were administration of corticosteroids within the previous 28 days, erythrocyte concentration in CSF greater than 500 per µL, and usage of disease-modifying MS therapy prior to sampling (MS patients only). Further exclusion criteria were presence of other inflammatory neurological diseases or active liver or kidney diseases, with known influence on the albumin redox state. 

### 4.2. Assessment of Routine Diagnostic Parameters in Serum and CSF

All CSF and serum samples were collected, processed, and stored according to international consensus guidelines [30]. Sampling of CSF and serum was performed in parallel, with 6–10 mL of CSF being obtained by lumbar puncture (LP) together with withdrawal of 8 mL of peripheral blood. Routine CSF diagnostic workup was performed as previously described [31]. Briefly, albumin and immunoglobulins (Ig) were determined by nephelometry on a Beckman Coulter Image 800 analyzer (Beckman Coulter Inc., Brea, CA, USA). Integrity of the blood CSF barrier was assessed by calculation of the CSF/serum ratio for albumin (Q_Alb_) [32]. Intrathecal IgG production was assessed quantitatively by calculation of the IgG index (reference < 0.7) [33] and qualitatively by looking for the presence of oligoclonal IgG bands (OCBs) using isoelectric focusing [34]. Routine diagnostic parameters are given together with albumin redox state values in Table 2. After routine diagnostic workup, samples were stored at −70 °C for a maximum of 16 months until HPLC analysis. 

### 4.3. Assessment of Clinical Parameters

Demographic and clinical parameters including age, sex, disease onset and duration, Expanded Disability Status Scale (EDSS) score, and annualized relapse rate were recorded by experienced neurologists at time of diagnosis and at scheduled clinical follow-up visits. A relapse was defined as a monophasic clinical episode with patient-reported symptoms and objective findings typical of multiple sclerosis, reflecting a focal or multifocal inflammatory demyelinating event in the CNS, developing acutely or subacutely, with a duration of at least 24 h, with or without recovery, and in the absence of fever or infection [28]. The EDSS is a 20-step scale used to classify the physical impairment of MS patients [35]. Demographic and clinical parameters of patients and controls are summarized in Table 1. The ‘disease duration’ was defined as the time (in months) between the first occurrence of clinical symptoms (=disease onset) and the day of sampling. The ‘EDSS in remission’ in RRMS patients was defined as the EDSS at scheduled control visits within 2 months of sampling, with either >28 days since onset of the last relapse or <28 days, but with already subsided symptoms. In PPMS, ‘EDSS in remission’ was defined as the EDSS at the closest control visit to sampling (maximum 177 days in between). 

### 4.4. Determination of Albumin Redox State in Serum and CSF

**Figure 4:**
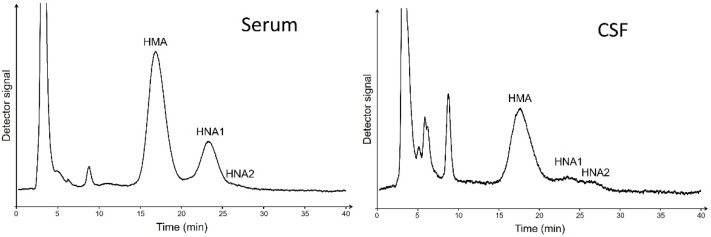
Exemplary HPLC chromatograms of albumin redox fractions in serum and CSF of one MS patient.

To determine the redox state of albumin in serum and CSF, albumin was separated into its different fractions (HMA, HNA1, and HNA2) by high-performance liquid chromatography (HPLC) as described by Hayashi et al. [36]. In brief, serum or CSF samples were diluted in 0.1 M sodium phosphate, 0.3 M sodium chloride, pH 6.87 (1:100 or 1:2, respectively), and filtered through a Whatman 0.45 µm nylon filter (Bartelt Labor- & Datentechnik, Graz, Austria). Diluted samples were stored in a Shimadzu SIL-20AC HT autosampler (Shimadzu HandelsgesmbH, Korneuburg, Austria) at 4 °C until injection of 20 µL of each diluted sample into the HPLC system. Separation of HMA, HNA1, and HNA2 was performed using a Shodex Asahipak ES-502 N 7C anion exchange column (7.5 × 100 mm, Bartelt Labor- & Datentechnik, Graz, Austria) with 50 mM sodium acetate, 400 mM sodium sulfate, pH 4.85, as mobile phase (A) and gradient elution was achieved with the same mobile phase containing 10% ethanol (*v*/*v*; mobile phase B) at a flow rate of 1 mL/min applied by an Hitachi Elite LaChrom gradient pump (Hitachi High-Tech Corporation). A linear gradient (5–25 min) of 0 to 60% mobile phase B and additional 5 min at 60% B was used for elution. The column was kept at 35 °C in a Shimadzu CTO-10AC VP column oven (Shimadzu). Detection was carried out by fluorescence at 280/340 nm with a Jasco 821FP fluorescence detector (Spectronex, Strasnice, Czech Republic) and quantification was based on the area of the individual peaks as determined using Peak Fit software (Version 4.12, SPSS Science, Chicago, IL, USA) by fitting Gaussian curves to the chromatograms. Examples of chromatograms obtained in serum and CSF of MS patients are shown in Figure 4.

### 4.5. Calculation of HMA, HNA1, and HNA2 Quotients

To estimate the ratio of reduced and oxidized albumin in CSF to serum we calculated HMA, HNA1, and HNA2 quotients (Q_HMA_, Q_HNA1_, Q_HNA2_). Absolute amounts of HMA, HNA1, and HNA2 in CSF and serum were calculated from the percentage of the respective albumin redox fraction and the total albumin concentration in CSF and serum, whereof the quotients were formed.

### 4.6. Statistics 

Results are presented as means ± SD or medians and interquartile ranges, depending on their distribution as assessed by Kolmogorov–Smirnoff test. Differences between the groups were assessed applying Student’s t-tests or Mann–Whitney U tests, as appropriate. Pearson or Spearman (partial) correlations between variables were performed. Differences are considered statistically significant for *p* < 0.05. Data were stored in a Microsoft Excel data sheet and statistical analyses were performed using GraphPad Prism (Version 9; GraphPad Software, San Diego, CA, USA) or IBM SPSS Statistics (Version 27, Armonk, NY, USA).

## Figures and Tables

**Table 1:** Demographic and clinical data of patients and controls.

	Patients (n = 20) RRMS/PPMS (n = 17/3)	Controls (n = 21)
n female	10	13
Age at sampling (years)	33 (27–46)	36 (29–42)
Age at disease onset (years)	32 (25–44)	n.a.
Disease duration (months)	1 (0–23)	n.a.
EDSS in remission	1.0 (0.0–2.0)	n.a.
	**RRMS (n = 17)**	
EDSS at last relapse	2.0 (1.0–2.5)	n.a.
n active disease ≤ 14 days prior to sampling	6	n.a.

Data are given at time of sampling and are presented as numbers or medians (25th–75th percentile). Differences between patients and controls regarding sex (*p* = 0.52) (by Fisher’s exact test) and age (*p* = 0.70) (by unpaired Student’s *t*-test) were not significant. EDSS, Expanded Disability Status Scale; n.a., not applicable.

**Table 2:** Routine diagnostic parameters and albumin redox state in serum and CSF of patients (RRMS and PPMS) and controls.

	Patients (n = 20)	Controls (n = 21)	*p*-Value
white cell count in CSF (n/µL)	7 (2–13)	1 (1–2)	<0.001
erythrocyte count in CSF (n/µL)	0 (0–10)	5 (0–51)	0.07
n oligoclonal bands positive	20	n.a.	n.a.
n BBB disruption ^#^	4	0	0.11
Lactate in CSF (mmol/L)	1.5 (1.4–1.6)	1.5 (1.3–2.6)	0.30
Total protein in CSF (mg/dL)	33 (26–38)	28 (24–33)	0.10
HSA Serum (g/dL)	4.56 (4.32–4.93)	4.51 (4.23–5.24)	0.16
HSA CSF (mg/dL)	21.6 (16.9–25.3)	21.7 (19.1–24.9)	0.92
QAlb (×10^−3^)	4.495 (3.623–5.793)	5.040 (3.790–5.525)	0.75
HMA Serum (%)	70.1 (66.9–72.7)	70.9 (66.3–73.2)	0.92
HNA1 Serum (%)	26.1 (24.3–29.4)	25.4 (24.0–30.1)	0.90
HNA2 Serum (%)	3.3 (2.9–3.7)	3.2 (3.0–3.8)	0.68
HMA CSF (%)	86.3 (82.3–90.0)	87.1 (84.8–90.1)	0.65
HNA1 CSF (%)	10.2 (8.8–14.3)	12.9 (9.5–14.0)	0.51
HNA2 CSF (%)	2.5 (0.0–4.7)	0.0 (0.0–2.5)	0.08

Data are given for time at sampling and are presented as number or as median (25th–75th percentile). Differences between variables of patients and controls were assessed by Mann–Whitney U test or Fisher’s exact test. Significant differences are highlighted in bold. ^#^, BBB disruption as evidenced by an increased CSF/serum albumin ratio; n.a., not applicable.

**Table 3:** Spearman correlations of albumin redox fractions in serum and CSF of patients (RRMS and PPMS) and controls with the age at sampling.

			Age at Sampling	
		Patients (n = 20)	Controls (n = 21)	Patients + Controls (n = 41)
HMA Serum	r	−0.311	−0.140	−0.231
	*p*	0.18	0.51	0.15
HNA1 Serum	r	0.329	0.064	0.213
	*p*	0.16	0.78	0.18
HNA2 Serum	r	0.241	0.605	0.417
	*p*	0.31	**0.004**	**0.007**
HMA CSF	r	−0.289	−0.399	−0.324
	*p*	0.22	0.07	**0.04**
HNA1 CSF	r	0.298	0.299	0.276
	*p*	0.20	0.19	0.08
HNA2 CSF	r	0.029	0.524	0.237
	*p*	0.90	**0.01**	0.14

Significant correlations are highlighted in bold.

**Table 4:** Non-parametric partial correlations of albumin redox fractions in serum with lactate in CSF of patients (RRMS and PPMS) and controls (control variable: age at sampling).

			Lactate in CSF	
		Patients (n = 20)	Controls (n = 21)	Patients + Controls (n = 41)
HMA Serum	r	−0.577	−0.399	−0.468
	*p*	**0.01**	0.08	**0.002**
HNA1 Serum	r	0.489	0.373	0.432
	*p*	**0.03**	0.11	**0.005**
HNA2 Serum	r	0.638	0.319	0.432
	*p*	**0.003**	0.17	**0.005**

Significant correlations are highlighted in bold.

## Data Availability

The data presented in this study are available from the corresponding authors upon reasonable request.
